# Ethnomedicinal plants used for malaria treatment in Rukungiri District, Western Uganda

**DOI:** 10.1186/s41182-023-00541-9

**Published:** 2023-08-30

**Authors:** Hannington Gumisiriza, Eunice Apio Olet, Paul Mukasa, Julius B. Lejju, Timothy Omara

**Affiliations:** 1 Department of Chemistry, Mbarara University of Science and Technology, P.O. Box 1410, Mbarara, Uganda; 2 Department of Biology, Mbarara University of Science and Technology, P.O. Box 1410, Mbarara, Uganda; 3 Department of Chemistry and Biochemistry, School of Sciences and Aerospace Studies, Moi University, P.O. Box 3900, Eldoret, Kenya; 4 Center of Excellence II in Phytochemicals, Textile and Renewable Energy (ACE II PTRE), Moi University, P.O. Box 3900, Eldoret, Kenya; 5 Present Address: Department of Chemistry, Institute of Chemistry of Renewable Resources, University of Natural Resources and Life Sciences, Vienna (BOKU), Konrad-Lorenz-Straße 24, 3430 Tulln, Austria

**Keywords:** African traditional medicine, Antimalarial resistance, Ethnobotanical knowledge, Malaria, Medicinal plants

## Abstract

**Background:**

Malaria remains a major global health challenge and a serious cause of morbidity and mortality in sub-Saharan Africa. In Uganda, limited access to medical facilities has perpetuated the reliance of indigenous communities on herbal medicine for the prevention and management of malaria. This study was undertaken to document ethnobotanical knowledge on medicinal plants prescribed for managing malaria in Rukungiri District, a meso-endemic malaria region of Western Uganda.

**Methods:**

An ethnobotanical survey was carried out between May 2022 and December 2022 in Bwambara Sub-County, Rukungiri District, Western Uganda using semi-structured questionnaire. A total of 125 respondents (81 females and 44 males) were randomly selected and seven (7) key informants were engaged in open interviews. In all cases, awareness of herbalists on malaria, treatment-seeking behaviour and herbal treatment practices were obtained. The ethnobotanical data were analyzed using descriptive statistics, informant consensus factor and preference ranking.

**Results:**

The study identified 48 medicinal plants belonging to 47 genera and 23 families used in the treatment of malaria and its symptoms in the study area. The most frequently cited species were *Vernonia* *amygdalina*, *Aloe* *vera* and *Azadirachta* *indica*. Leaves (74%) was the most used plant organ, mostly for preparation of decoctions (41.8%) and infusions (23.6%) which are administered orally (89.6%) or used for bathing (10.4%).

**Conclusions:**

Indigenous knowledge of medicinal plants used as prophylaxis and for treatment of malaria still exist among the local communities of Bwambara Sub-County. However, there is a need to investigate the antimalarial efficacy, phytochemical composition and safety of species (such as *Digitaria* *abyssinica* and *Berkheya* *barbata*) with high percentage use values to validate their use.

**Supplementary Information:**

The online version contains supplementary material available at 10.1186/s41182-023-00541-9.

## Background

Malaria has afflicted humanity for millennia [1]. It is one of the most fatal, preventable and curable parasitic diseases globally, with about 619,000 deaths and 247 million new cases reported in 2021 [2]. In the WHO African Region, 593,000 malaria deaths were reported in 2021 [2]. Recent estimates indicate that almost half of the global population live in 82 malaria-endemic countries [3]. The majority of malaria deaths are common in the tropical and subtropical regions, particularly Central, Western and East Africa, where there is limited healthcare and/or vector (female *Anopheles* mosquito) control [4].

According to World Malaria Report 2022 [2], about 96% of the global malaria-related deaths were reported in 29 countries. Uganda has one of the highest recorded malaria transmission rates on the African continent, with over 90% of the population at risk [5]. Malaria has also remained the major cause of morbidity and mortality in Uganda, as evidenced by 30–50% of outpatient visits and 15–20% of hospital admissions. The average economic loss in Uganda due to malaria is more than US$ 500 million per year [6]. In 2021, the World Health Organization (WHO) estimated that there were 12.4 million malaria cases and over 31,350 malaria deaths in Uganda [2]. In 2022, the Ugandan Ministry of Health indicated that 68 districts recorded malaria upsurge [7]. Rukungiri District recorded 196,070 malaria cases and some deaths between 25th April and 1st May 2022, where Bwambara Sub-County (the focal area of this study) accounted for all the malaria deaths within the whole district [7]. This could be due to the poor public healthcare system in the Sub-County [8].

Inefficient malaria treatment has promoted antimalarial drug resistance and this has been cited to be of considerable concern in East Africa [2]. In Uganda, artemisinin resistance by *Plasmodium* *falciparum* was identified, where two pathogenic mutations were found in more than 15% of the collected samples [9]. The WHO calls for response to the emergence of antimalarial drug resistance by ensuring that efficacious treatments remain available. This calls for alternative interventions for malaria treatment which include use and exploration of medicinal plants [2].

Medicinal plants contain phytochemicals with intriguing biological activities which provide prospects for new drug discoveries [10]. Plants have remained the focus of many studies aiming at discovering antimalarials, because the current conventional antimalarial drugs such as artemisinin and quinine were discovered from *Artemisia* *annua* and *Cinchona* species [11]. In this context, documentation of medicinal plants used in the treatment of malaria constitutes a vital task in the preservation of indigenous knowledge and biodiversity, as well as improvement of malaria treatment interventions amongst communities. It also contributes to bioprospecting, which lays a foundation for further research on the safety and efficacy of medicinal plants, and identification of bioactive compounds that could act as druggable hit molecules for the development of new antimalarial therapies.

Previous studies in Rukungiri District have documented the ethnomedicinal plants used for managing pediatric diseases [12] and "African" diseases [13]. Based on the latter report, we found that indigenous communities of Bwambara Sub-County (Rukungiri District) cherish medicinal plants and knowledge of their utilization, management and conservation. We identified one of the most used species in this study area (*Gouania* *longispicata* Engl.) and reported on its antimicrobial activity [14]. Despite the floral biodiversity and rich ethnocultural heritage of Bwambara Sub-County [15] and Rukungiri District as a whole, there is no report on medicinal plants used in malaria prevention and treatment. Coupled with the rapid loss of vegetation and adoption of foreign cultures by the local societies [16], there is a pressing need to record such indigenous knowledge before it is lost forever. Thus, this study documented the ethnobotanical knowledge on medicinal plants that are used for treatment of malaria in Bwambara Sub-County, Rukungiri District, Western Uganda.

## Materials and methods

### Study area

**Fig. 1:**
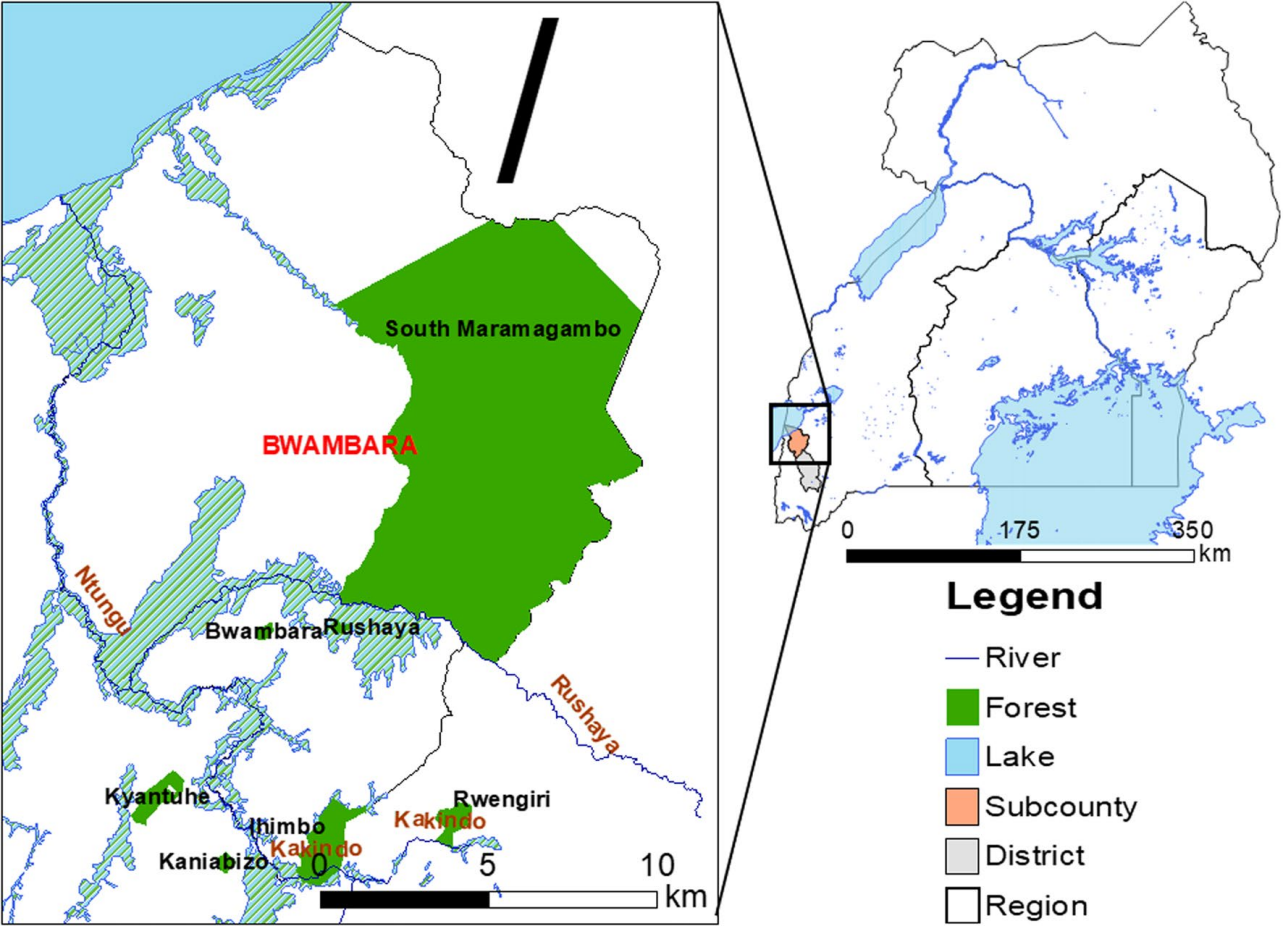
Map of Rukungiri district, Western Uganda showing the location of the study area (Bwambara Sub-County)

This study was conducted in Bwambara Sub-County (latitudes: − 0.33778 and − 0.67119, and longitudes: 29.69882 and 29.86939), Rukungiri District, Western Uganda (Fig. 1). The area is dominated by the Bakiga ethnic group, but other ethnic groups such as the Bahororo and Banyabutumbi are also represented. The main occupation is subsistence farming, except in Rwenshama Parish, where fishing is the main economic activity [13]. The major crops grown are coffee, matooke, grapes, pears and peaches, while cattle are majorly kept for milk production [17]. Bwambara Sub-County is bordered by Lake Edward, Queen Elizabeth National Park and Kigezi Game Reserve [18].

The area has forests, forest/savannah mosaic and savannah type of vegetation [19]. It experiences a bimodal rainfall, with a short rainy season from March to May and a long rainy season between September and December. Thus, malaria transmission is perennial with maximum prevalence shortly after the rainy seasons [8, 20] and is, therefore, meso-endemic for malaria with moderate-to-high transmission [21]. The public healthcare system in the Sub-County has four health centres that serve its 79 villages with an estimated population of 28,900 people [8]. Two are Public Health Centre II but only limited to outpatient services. The remaining two are Public Health Centre III facilities, which in addition to outpatient services, offer maternity services, has an inpatient ward and microscopy for diagnosing malaria. However, these health centres have inadequate facilities, are poorly stocked and have few health workers [18]. Majority of the population are unable to access good services at the health centres due to long distances, unavailability of medicine, poverty and neglect by medical staff.

### Sample size determination

The calculated sample size (*S*) was 125 respondents, obtained using the formula suggested by Krejcie and Morgan [22], that is

$$S = X^{2} NP\left( {1 - \, P} \right) \div d^{2} \left( {N - 1} \right) + X^{2} P \, \left( {1 - \, P} \right)$$

where *S*  is  the required sample size, *X*^2^ = the table value of chi-square for 1 degree of freedom at the desired confidence level (3.841), *N* = the population size, *P* = the population proportion (assumed to be 0.50, since this would provide the maximum sample size) and *d* = the degree of accuracy expressed as a proportion (0.05).

### Ethnobotanical survey

A cross-sectional survey using semi-structured questionnaires was undertaken between May 2022 and December 2022. With the help of local council representatives, seven specialized herbalists known to treat patients using herbal medicine were purposively sampled and interviewed as key informants. The study was conducted in *Rukiga/Runyankole*, the commonly used local languages in the area. The questions mainly focused on how informants prevented and diagnosed malaria, and how medicinal plants were prepared and administered for treatment of malaria.

Voucher specimens were prepared for all the collected plant species and deposited at the Department of Biology, Mbarara University of Science and Technology (Uganda), where they were authenticated by a botanist. The scientific plant names were given according to The World Flora Online (http://www.worldfloraonline.org) and International Plant Name Index (www.ipni.org). The family names of the plant species were cross-checked with the Angiosperm Phylogeny Group (www.gbif.org).

### Data analysis

Numerical data were captured in Microsoft Excel for Windows (Microsoft Corporation, USA) and thereafter exported to SPSS software (v26, SPSS Inc., USA) for analysis. Qualitative data on malaria prevention, ethnobotanical knowledge and respondents’ socio-demographic characteristics were analyzed using descriptive statistics, such as percentages and frequencies.

Quantitative ethnobotanical data were used to calculate frequency of citation (FC) and informant consensus factor (ICF). The FC was used to assess the number of informants who were familiar with usage of a particular medicinal plant to treat malaria. Use report (UR) was recorded whenever any informant mentioned use of a medicinal plant in a particular way. The ICF was used to determine the homogeneity of the collected ethnobotanical data using the formula [23]:

$${\text{ICF}} = \left( {N_{{{\text{ur}}}} {-}N_{{\text{t}}} } \right)/\left( {N_{{{\text{ur}}}} {-} \, 1} \right)$$

where *N*_ur_ = total number of use reports for each disease category, and *N*_t_ = total number of species in each use category. The ICF value ranges from 0 to 1. A high ICF value (close to 1) signifies that a high proportion of informants uses a few plant species to treat a particular disease, and this suggests that the community possesses a well-defined mechanism for exchange of indigenous knowledge. Conversely, low ICF values (close to 0) indicate that many plant species are reported to be used by a high proportion of informants to treat a particular disease, implying that there is lack of information exchange within the community or the medicinal plants are randomly selected [24].

## Results

### Socio-demographic characteristics

The study involved 125 informants, of which there were 64.8% females and 35.2% males (Table 1). The largest number of respondents were 31–40 years (27.8%). In general, 53.5% were above 40 years, while only 18.5% were ≤ 30 years. The findings revealed that most of the informants had attended primary school (55.6%), while 30.4% did not attain any formal education. The main religion in the area is Anglican (45.6%) and Catholic (31.6%).

**Table 1:** Socio-demographic characteristics of informants from Bwambara Sub-County, Rukungiri District, Uganda

Variable	Characteristic	Percentage (%)
Gender	Male	35.2
	Female	64.8
Age group (years)	18–19	0.8
	20–30	17.7
	31–40	27.8
	41–50	21.5
	51–60	15.2
	> 60	17
Educational status	None	30.4
	Primary	55.6
	Secondary	13.2
	College	0.8
Religious affiliation	Anglican	45.6
	Catholic	31.6
	Pentecostal	16.5
	Moslem	6.3
Ethnic group	Bakiga	83.5
	Bahororo	5.1
	Banyabutumbi	3.8
	Banyankole	2.5
	Banyarwanda	0.8
	Congolese	0.8
Occupation	Peasant farmer	82.3
	Business	10.1
	Teacher	0.8
Income (UgX)	None	31.6
	< 50,000	27.8
	50,001–80,000	19
	> 80,000	12.7

*UgX*  Ugandan shillings, and 1 US$ = 3827 UgX

Approximately 31.6% of the respondents declared a lack of monthly (source of) income, while 27.8% others earned less than 50,000 Uganda shillings (UgX) (US$ 13.07) per month. With the exception of a formally employed primary school teacher who receive monthly salary, the rest got their earnings from harvesting crops. The monthly earnings were got from the sum of the block money earned after selling the harvests at the end seasons divided by 12 months. Therefore, the locals hardly had any income outside harvesting periods. Moreover, most locals carried out subsistence farming, where food is simply grown for consumption. The highest monthly income was UgX 560,000 (US$ 146.33), earned by the primary school teacher.

### Source of knowledge of medicinal plants

**Fig. 2:**
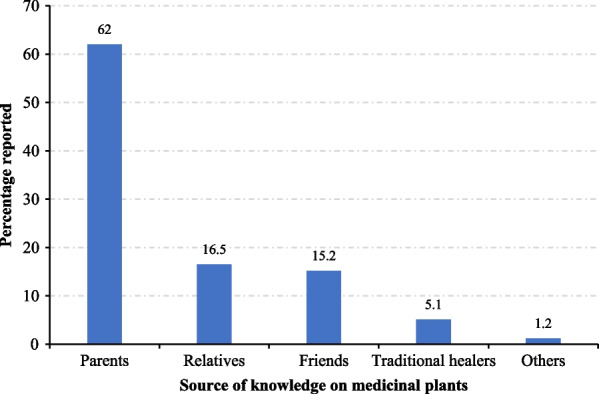
Source of knowledge on plants used for malaria treatment in Bwambara Sub-County, Rukungiri District, Uganda

Majority of the respondents (62%) acquired knowledge on ethnomedicinal plants for malaria treatment from their parents, while 16.5% got knowledge from older family members, usually grandparents (Fig. 2). Traditional healers contributed 5.1% to the transfer of indigenous knowledge. However, a very small portion of the respondents acquired ethnobotanical knowledge through other means, such as spiritual revelations, radio and television programs, and reading ethnobotanical books.

### Symptoms of malaria and treatment-seeking behaviour of patients

In the study area, malaria is locally known as *Omushweija* *gw’ensiri* (*Omushweija* means fever, *ensiri* means mosquito). Thus, this is directly translated to mean “fever of mosquitoes”. For this, all informants were aware that malaria is caused by mosquito bites. Some informants reported that drinking unboiled water, exposure to morning dew and rain may cause malaria. Other indirect causes of malaria such as having grown bushes around homesteads, leaving the house doors and windows open in the evening hours and having stagnant water were also cited.

The most important symptoms of malaria were feeling cold (having goosebumps), high body temperature, headache, shivering, vomiting and joint pains (Table 2). It should be noted that all or a combination of the listed symptoms may be present in a malaria patient. Thus, the informants diagnose a certain disease as malaria if the patient presented with at least three of the listed symptoms. The participants were aware that not all patients recover from malaria after taking herbal remedies, which necessitated referral to hospitals. Participants reported that referrals are made basing on severity of the symptoms (i.e., extremely high body temperature and confusion) (Table 3).

**Table 2:** Malaria symptoms mentioned by respondents in Bwambara, Rukungiri District, Uganda (*n* = 125)

General malaria symptoms	Local language (Runyankore/Rukiga)	Percentage
Feeling cold/goosebumps	Okupiipa	82
High temperature	Omuriro	79
Headache	Okuterwa omutwe	77
Shivering	Okutetema	75
Vomiting	Okutanaka	72
Joint pains	Okushaash engyingo	70
Loss of appetite	Okuremwa kurya	58
General body weakness	Okubura amaani	42
Abdominal pain	Okushaasha omunda	37
Wound or burns	Ebironda/amasya	26
Sweating	Okututuuka	23
Sticky or red eyes	Okutukura amaisho	17
Bitter or sour mouth	Okushariira akanwa	15
Anemia-very pale palms, fingernails	Okuhwamu eshagama–okwera engaro n’enono	13
False smell	Okunukirwa ebitariho	12
Flue	Sanyiga	11
Diarrhea	Okwirukana	10
Frequent thirst	Okugyira eiriho buri kanya	9
Teary eyes	Okurira amaisho	8
Severe dehydration indicated by pale eyes	Okuhwamu amaizi–amaisho garikwera	7
Yellow eyes	Amaisho g’akinekye	7
Difficulty in breathing	Okwisya kubi	2
Painful swelling or lumps in the skin	Okuzimba	2

**Table 3:** Severe malaria symptoms that warrant hospitalization among indigenous communities in Bwambara Sub-County, Rukungiri District, Western Uganda

Severe symptoms	Informants							Total	Ranking
	*I*_1_	*I*_2_	*I*_3_	*I*_4_	*I*_5_	*I*_6_	*I*_7_		
Coma/loss of consciousness	5	4	3	5	4	4	5	30	3rd
Confusion	3	7	5	6	6	5	6	38	2nd
Convulsions	6	3	7	3	3	6	1	29	4th
Diarrhea	1	2	1	4	1	1	2	12	7th
Extremely high body temperature	7	6	6	7	7	7	7	47	1st
Fits in children	4	5	4	3	5	3	4	28	5^th^
Vomiting—cannot keep any food or drink	2	1	2	1	2	2	3	13	6^th^

*I*  Informant

### Prevention of malaria

**Fig. 3:**
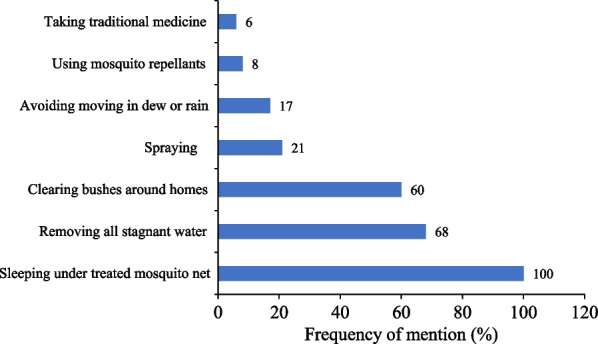
Measures used for malaria prevention in Bwambara Sub-County, Rukungiri District, Western Uganda

All the informants reported that sleeping under treated mosquito net prevents contracting malaria. Other preventive measures included destruction of mosquito breeding areas such aas stagnant waters and elimination of unwanted vegetation within and around homesteads (Fig. 3).

### Diversity of ethnomedicinal plants used for malaria treatment

A total of 55 plant species were mentioned to be used for treatment of malaria (Table 4). However, only 48 of the voucher specimens collected were taxonomically identified. The other seven species could not be found within the study area during field visits, and only their local names were recorded (Table 4). The identified species belonged to 47 genera and 23 families. Most plant species belonged to family Asteraceae (22.9%), Fabaceae (10.4%), Lamiaceae (8.3%) and Poaceae (8.3%). The calculated ICF value for malaria was 0.81. Of the medicinal plants reported for malaria treatment, a paired comparison indicates that the highest rank was given for *Vernonia* *amygdalina* (FC = 76; UV = 0.608), followed by *Aloe* *vera* (FC = 35; UV = 0.28) and then *Azadirachta* *indica* (FC = 15; UV = 0.12).

**Table 4:** Medicinal plants used in the management of malaria in Bwambara Subcounty, Rukungiri District, Uganda

No.	Family *Botanical* *name*	Voucher number	Part used	Life form	FC	UV
	Acanthaceae					
1	*Justicia* *anselliana* (Nees) T. Anderson	H22-013	L	Shrub	1	0.008
	Apocynaceae					
2	*Mondia* *whitei* (Hook.f.) Skeels	H22-030	L	Vine	1	0.008
3	*Cascabela* *thevetia* (L.) Lippod	H22-049	L	Tree	1	0.008
	Asphodelaceae					
4	*Aloe* *vera* (L.) Burm.f	H22-022	L	Herb	35	0.28
	Asteraceae					
5	*Baccharoides* *lasiopus* (O.Hoffm) H.Rob	H22-008	L, RB	Shrub	6	0.048
6	*Berkheya* *barbata* (L.f.) Hutch	H22-040	L	Herb	1	0.008
7	*Bidens* *pilosa* L	H22-035	Fl	Herb	3	0.024
8	*Bothriocline* *longipes* (Oliv. & Hiern) N.E.Br	H22-046	L	Herb	1	0.008
9	*Crassocephalum* *vitellinum* (Benth.) S. Moore	H22-016	L	Herb	1	0.008
10	*Vernonia* *amygdalina* Delile	H22-002	L, RB	Shrub	76	0.608
11	*Hoffmannanthus* *abbotianus* (O.Hoffm.) H.Rob., S.C.Keeley & Skvarla	H22-019	L	Herb	4	0.032
12	*Laggera* *alata* (D.Don) Sch.Bip. ex Oliv	H22-020	L	Herb	1	0.008
13	*Solanecio* *mannii* (Hook.f.) C. Jeffrey	H22-005	L	Shrub	2	0.016
14	*Sonchus* *oleraceus* L	H22-011	St	Herb	5	0.04
15	*Tithonia* *diversifolia* (Hemsl.) A.Gray	H22-050	L	Shrub	5	0.04
	Bignoniaceae					
16	*Kigelia* *africana* (Lam.) Benth	H22-036	L	Tree	1	0.008
17	*Markhamia* *lutea* (Benth.) K.Schum	H22-031	RB	Tree	1	0.008
	Canellaceae					
18	*Warburgia* *ugandensis* Sprague	H22-014	SB	Tree	3	0.024
	Caricaceae					
19	*Carica* *papaya* L	H22-047	SB, L, Fr	Tree	6	0.048
	Cleomaceae					
20	*Cleome* *gynandra* L	H22-001	L	Herb	1	0.008
	Cucurbitaceae					
21	*Momordica* *foetida* Schumach	H22-025	L, SB	Vine	2	0.016
	Euphorbiaceae					
22	*Manihot* *esculenta* Crantz	H22-033	L	Shrub	1	0.008
23	*Shirakiopsis* *elliptica* (Hochst.) Esser	H22-009	L	Tree	1	0.008
	Fabaceae					
24	*Albizia* *coriaria* Welw. ex Oliv	H22-012	SB	Tree	2	0.016
25	*Erythrina* *abyssinica* Lam	H22-010	L	Tree	3	0.024
26	*Indigofera* *arrecta* Hochst. ex A.Rich	H22-006	L, RB	Shrub	1	0.008
27	*Senna* *didymobotrya* (Fresen.) H.S. Irwin & Barneby	H22-037	L	Shrub	2	0.016
28	*Vachellia* *hockii* (De Wild.) Seigler & Ebinger	H22-041	SB	Tree	1	0.008
	Lamiaceae					
29	*Clerodendrum* *capitatum* (Wild.) Schumach	H22-021	L	Shrub	5	0.032
30	*Ocimum* *gratissimum*	H22-003	L	Shrub	1	0.008
31	*Ocimum* *kilimandscharicum* Gürke	H22-018	L	Shrub	2	0.016
32	*Plectranthus* *barbatus* Andrews	H22-007	L	Shrub	3	0.024
	Malvaceae					
33	*Sida* *alba* L	H22-044	L	Shrub	3	0.024
	Meliaceae					
34	*Azadirachta* *indica* A. Juss	H22-048	L	Tree	15	0.12
	Moringaceae					
35	*Moringa* *oleifera* Lam	H22-027	Seed, L	Tree	4	0.032
	Musaceae					
36	*Musa* *acuminata* Colla	H22-023	L	Herb	1	0.008
	Myrtaceae					
37	*Eucalyptus* *grandis* W. Hill ex Maiden	H22-039	L	Tree	2	0.016
	Poaceae					
38	*Digitaria* *abyssinica* (Hochst. ex A.Rich.) Stapf	H22-026	L	Grass	6	0.048
39	*Imperata* *cylindrica* (L.) P.Beauv	H22-042	L	Grass	1	0.008
40	*Pennisetum* *purpureum* Schumach	H22-029	St	Grass	2	0.016
41	*Saccharum* *officinarum* L	H22-004	St	Grass	1	0.008
	Rhamnaceae					
42	*Gouania* *longispicata* Engl	H22-028	L	Liana	1	0.008
	Rutaceae					
43	*Citrus* *limon* (L.) Burm.fil	H22-043	L, SB	Tree	2	0.016
44	*Clausena* *anisata* (Willd.) Hook.f	H22-032	L	Shrub	1	0.008
	Solanaceae					
45	*Nicotiana* *tabacum* L	H22-015	L	Herb	1	0.008
46	*Physalis* *peruviana* L	H22-034	L, RB	Herb	3	0.024
	Verbenaceae					
47	*Lantana* *trifolia* L	H22-024	L	Shrub	3	0.024
	Vitaceae					
48	*Cyphostemma* *adenocaule* (Steud. ex A.Rich.) Desc	H22-017	L	Liana	1	0.008
49	Unidentified	NA	L	NA	6	0.048
50	Unidentified	NA	L	NA	3	0.024
51	Unidentified	NA	L	NA	1	0.008
52	Unidentified	NA	L	NA	2	0.016
53	Unidentified	NA	L	NA	4	0.032
54	Unidentified	NA	L	NA	1	0.008
55	Unidentified	NA	L	NA	1	0.008

*NA*  not applicable, *FC*  frequency of citation, *UV*  use value, plant parts: *Bk*  bark, *Fl*  flowers, *Fr*  fruits, *L*  leaf, *RB*  root bark, *St*  stem, *SB*  stem bark

### Life forms, plant parts, preparation and administration of herbal remedies for malaria treatment

**Fig. 4:**
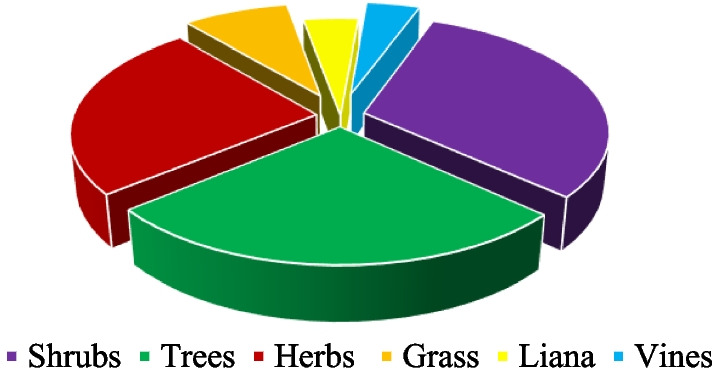
Life forms of medicinal plants used in the management of malaria in Bwambara Sub-County, Rukungiri District, Western Uganda

**Fig. 5:**
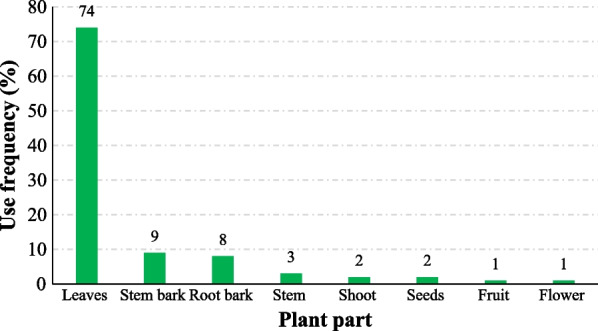
Plant parts used in preparation of remedies for malaria treatment in Bwambara Sub-County, Rukungiri District, Western Uganda

The identified plant species consisted of mainly shrubs (31.3%), trees (27.1%) and herbs (25.0%) (Fig. 4; Additional file 1). On the other hand, the most used plant organs were leaves (74%), followed by stem bark (9%) and root bark (8%) (Fig. 5). Most of the plants were used in combination with each other (65.5%). All the plant materials were used immediately after collection (while still fresh). Only the stem bark of *Warburgia* *ugandensis* and leaves of *Azadirachta* *indica* were reported to be used either when fresh or in powder form.

The most common preparation methods were decoctions (41.8%) and cold infusion (23.6%) (Additional file 1). Other methods included bathing (3.6%) and steaming (3.6%). Some herbalists cited continuous use of some medicinal plants such as *Azadirachta* *indica* as tea or in tea as a prophylaxis for malaria.

## Discussion

This study showed that most of the respondents were females (Table 1). This observation is contrary to a recent report by Tabuti et al. [24] in Eastern Uganda. Previous studies in Uganda have indicated higher participation of females than males in ethnobotanical surveys, since they are the ones who take healthcare needs of their families [25]. Moreover, more females could have participated because of their indigenous knowledge of nutri-medicinal plants as home managers and disease managers amongst children [26, 27]. Most respondents were the Bakiga, because they were the first group of Bantu ethnic group to migrate into this area as early as the 1950s, where the original inhabitants were the minority Banyabutumbi [28]. There were low-income levels among the respondents indicating that most of them depended on subsistence farming. This is typical in Uganda, and may also explain their preference of medicinal plants over conventional antimalarial drugs.

Regarding medicinal plant knowledge dynamics, most respondents hinted that they inherited the indigenous knowledge about medicinal plants for malaria treatment from their parents or grandparents (Fig. 2). This is not surprising, because African traditional medicine recipes are usually a guarded family secret, so that they are only passed orally to children from parents or through apprenticeships from relatives [16]. It was interesting to note that traditional healers contributed 5.1% to the transfer of indigenous knowledge (Fig. 2). This may be attributed to the cultural acceptability and strong belief in herbal medicines which has driven commercialization of herbal products. Therefore, the impetus behind acquiring herbal medicine knowledge observed in this study may be related to the need to establish a source of income through sale of herbal products [29]. Absence of a respondent who had acquired folk knowledge through formal training is because herbal medicine training centres are rare in Uganda.

The indigenous communities surveyed were well-informed that malaria is caused by mosquito bites, though some believed that taking unboiled water, exposure to morning dew as well as rain are other potential causes. Other indirect causes of malaria such as having grown bushes around homesteads, leaving the house doors and windows open in the evening hours and having stagnant water were also cited. A previous study in Uganda by Adia et al. [25] reported that 96% of the respondents understood that malaria was caused by infected mosquito bites. In similar study in Zimbabwe, all respondents recognized mosquito bites as the main cause of malaria [30] but traditional healers still held it that exposure to morning dew and drinking water from unprotected wells caused malaria. In Kenya, it was cited that malaria is believed to be a direct cause of mosquito bites, exposure to cold weather [31] or consumption of fresh unboiled milk (*Cheko* *che* *makiyo* in the Tugen dialect), dirty water, *ikwek* (i.e., vegetables, such as *Gynadropis* *gynadra* and *Solanum* *nigrum*) [32], witchcraft (sorcery attacks) and supernatural forces [33, 34]. In Eastern Uganda, some people believe that keeping a dirty homestead with dense bushes or pools of stagnant water caused malaria [35]. Though bushes and stagnant water may not be the direct causes of malaria, they are breeding sites for mosquitoes which transmit malaria. In this case, some communities would close windows and doors in the evening to guard against mosquito bites [35].

The most important symptoms of malaria were feeling cold/goosebumps, high body temperature, headache, shivering, vomiting and joint pains. These findings are consistent with Stangeland et al. [36] who reported high body temperature, shivering and headache as the most frequent symptoms of malaria in Nyakayojo Sub-County, Western Uganda. Another study in Central Uganda mentioned high body temperature, red eyes and vomiting as the most common malaria symptoms [25]. In the malaria-endemic Eastern Uganda, fever was mentioned as the major malaria symptom, along with vomiting, body weakness, headache, diarrhea, convulsions, inappetence and body chills [24]. Outside Uganda, malaria has been associated with periodic fever, sweating, backache, chills, headache, vomiting, diarrhea and joint pains [37, 38], coldness, goose pimples and headaches [30], which aligns well with our findings.

As indicated by the herbalists, some severe malaria symptoms such as very high body temperatures and confusion (probably due to cerebral malaria) were indicative that the patient needed to be referred for further treatment using conventional medicine (Table 3). This suggests that most people seek for conventional medical treatment when malaria is in severe stages. The listed symptoms relate with those of cerebral malaria, which may signify its prevalence in the study area. A study by Stangeland et al. [36] in Western Uganda found that hospital referrals were being recommended only in cases, where herbal remedies did not relieve malaria symptoms within 2–3 days of treatment or if the patient presented severe symptoms. The current findings agree with previous studies among traditional healers which reported few hospital referrals in exceptional cases when patients showed severe malaria symptoms or herbal remedies failed completely [24, 30, 37]. In a study by Ngarivhume et al. [30], lack of a patient’s improvement was attributed to evil spirits, where the patient needed cleansing/placating the spirits and then repeat or be given a different prescription thereafter. Such practices have been cited in Kenya, where malaria which is thought to be caused by supernatural forces required the intervention of diviners (such as *Oloiboni* among the Maasai and *Orgoiyon* among the Tugen) [33]. Such practices and beliefs, coupled with improper diagnosis and wrong choice of appropriate herbal remedies may result in death. This requires sensitization of the population about proper diagnosis of malaria and the dangers associated with late treatment.

All the informants reported that sleeping under treated mosquito net prevents contracting malaria (Fig. 3). This could be because since November 2018, Uganda as part of the eleven (high burden to high impact) countries distributed insecticide-treated mosquito nets (ITN) across the country [39]. This countrywide campaign could be the reason as to why each informant knew about ITN. Other preventive measures included the destruction of mosquito breeding areas, such as stagnant waters, and the reduction of unwanted vegetation within and around the homesteads. In Uganda, Tabuti [35] and Adia et al. [25] reported similar preventive measures used to guard against malaria. These findings are comparable to that in Zimbabwe where the use of ITN, destruction of mosquito breeding and resting areas, and reduction of unwanted undergrowth and puddles within and around homesteads, avoiding dew and residual spraying as malaria preventive measures are used to prevent malaria [30].

We identified 48 species, out of the 55 species mentioned by the respondents. The other species which could not be found during the field visits suggest that some plants are rare in the study area, or may have become extinct due to over exploitation or habitat destruction [18]. These raises need for instituting conservation measures. The identified plant species were majorly members of family Asteraceae, Fabaceae, Lamiaceae and Poaceae, which is in agreement with studies in Uganda [24, 25, 36, 40] and elsewhere [37, 41]. The prevalence of these families may be attributed to the frequent distribution in the area, diverse habitat, availability and presence of diverse secondary metabolites which increase the effectiveness of herbal remedies from them [42].

Of the 48 plants mentioned by herbalists, most species were listed for use in the management of malaria in Uganda and other countries. For instance, *Carica* *papaya*, *Albizia* *coriaria*, *Warburgia* *ugandensis*, *Azadirachta* *indica*, *Aloe* *vera* and *Vernonia* *amygdalina* in Uganda [24, 25, 40, 43–45], Zimbabwe [30], Kenya [38], Cameroon [46], Indonesia [47] and Ethiopia [48]. The calculated ICF for malaria was 0.81, implying that there is substantial agreement amongst the local population about the medicinal plants used in malaria treatment.

Leaves of shrubs, trees and herbs are the commonly used parts for preparing malaria remedies, corroborating some ethnobotanical reports in other parts the country [24, 25, 35, 36]. This could translate into a more sustainable use of the plants, as compared to roots. Leaves regenerate under favorable conditions to preserve the occurrence of the plants but their high use frequency in this study may plausibly be related to their year-round availability [49]. The observed use of different plants or plant parts could be to exploit their synergistic therapeutic effects [36, 50, 51], mask toxicity of efficacious plants or as a trick of keeping the secrecy of herbal formulae [52].

Oral administration (89.6%) was the major route used, but use of prepared decoctions for bathing was also practiced (10.4%). This is expected, because oral dosage forms are easy to prepare and administer, especially because malaria is an internal disease [38, 53]. One striking feature was that posology of the remedies were based on cups or glasses, a phenomenon that has been cited among herbalists throughout the East African botanical plate [24, 38, 40].

An in-depth search of literature indicated that 22 species recorded in this ethnobotanical study have been evaluated for their antimalarial/antiplasmodial activities (Table 5). Most species possess acceptable preclinical efficacy and safety, with the exception of *Vernonia* *amygdalina* [54] and *Senna* *Didymobotrya* [55] which showed high toxicity. It is worth noting that *Azadirachta* *indica* and *Vernonia* *amygdalina* has been subjected to clinical trials. A homeopathic *Azadirachta* *indica* preparation was shown to be effective for reducing malaria attacks in a highly endemic area for *Plasmodium* *falciparum* [56]. Similarly, Challand and Willcox [57] performed a clinical trial utilizing a remedy of *Vernonia* *amygdalina* leaves for the treatment of uncomplicated malaria. They found that there was an adequate clinical response at day 14 in 67% of cases. Total parasite clearance was observed only in 32% of those with adequate clinical response, where recrudescence occurred in 71% and this hampers complete remission of the parasite from the body [57]. In conclusion, these reports support that the inventoried medicinal plants possess antimalarial properties which can be further investigated for development of new antimalarial drug candidates. Specifically, clinical trials should be done on some of the species that have exhibited promising antimalarial efficacy. However, these maybe impeded by the strict regulatory requirements for clinical studies, as well as the financial muscle required [38]. In addition, formulations rich in known bioactive compounds from these species should be specifically targeted.

**Table 5:** Antiplasmodial, antimalarial and toxicity studies of extracts and compounds isolated from plants reported in malaria phytotherapy of Bwambara Sub-County, Rukungiri District, Western Uganda

Medicinal plant	Plant organ	Solvent^1^	Antiplasmodial/antimalarial (*Plasmodium* spp) efficacy^2^	Reported bioactive phytochemicals	Toxicity profile	References
*Aloe* *vera*	Leaves	Water	EC_50_ values for extract: 0.289 to 1056 μg/mL (MRC-2), 169.76 (3D7) for aloin	Aloin	Considered to be safe	[58, 59]
*Albizia* *coriaria*	Stem bark	MeOH	15.2 (D6); 16.8 (W2)	Triterpenoids, lupeol, lupenone	Cytotoxic to the human glioblastoma cell line U87 CD4 CXCR4 (​​CC_50_ = 6.4 and 14.9 μg/ml for ethanol and DMSO extracts)	[60–63]
*Azadirachta* *indica*	Leaves	Water, MeOH	17.9 (D6); 43.7 (W2)	Terpenoids, isoprenoids, gedunin, limonoids: khayanthone, meldenin, nimbinin	Cytotoxicity LD_50_ of 101.26 and 61.43 µg/ml for water and methanol extracts	[64–68]
*Bidens* *pilosa*	Leaves	DCM, water, MeOH	8.5, 5, 11, 70 (D10)	No reports	Hydro and ethanolic extracts are not toxic in mice (LD_50_ = 12.3 and 6.2 g/kg bw, respectively). Safe in humans	[69–71]
*Carica* *papaya*	Leaves	Ethyl acetate	2.96 (D10), 3.98 (DD2), 0.2 µM (carpaine)	Carpaine	Carpaine has high selectivity (106), nontoxic to normal red blood cells and rat skeletal myoblast (L6) cells	[72–74]
*Clausena* *anisata*	Twigs, Leaves	DCM/MeOH, water	18 (D10), > 100 (D10); 55, > 100 (D10)	No reports	Potentially toxic	[69, 75]
*Cleome* *gynandra*	Whole plant	Hexane, ethyl acetate, MeOH	Schizonticidal activity in vitro (NK65)	Apigenin, caffeic, Ferulic, hexadecanoic acids, kaempferol, taraxasterol, pheophytin A, sitosterol, stigmasterol; 5,7,13,15-eicosatetraen-9, 12-diol and 9-hydroxy-5,7,13,15-eicosatetraen-12-one	No report	[76]
*Clerodendrum* *capitatum*	Leaves	Ethanol	65.3% chemosuppression at 400 mg/kg	Tannins, flavonoids, alkaloids, triterpenes	LD_50_ > 5000 mg/kg	[77]
*Erythrina* *abyssinica*	Stem bark	Ethyl acetate	83.6% inhibition of *P.* *falciparum* at 10 μg/ml	Chalcones (5-prenylbutein, homobutein), flavanones such as 5-deoxyabyssinin II, abyssinin III and abyssinone IV	Minimal toxicity reported in animal studies	[54, 78]
*Kigelia* *africana*	Bark, fruit	Chloroform/ethyl acetate, MeOH	59.9 (K39), 83.8 (V1/S); fruits had 165.9 (K39)	None reported	Aqueous fruit extract elicited hepatorenal toxic effects in rats	[79, 80]
*Markhamia* *lutea*	Leaves	Ethyl acetate	71% inhibition of *P.* *falciparum* at 10 μg/ml	Phenylpropanoid glycosides, cycloartane triterpenoids, musambins A-C, Candmusambiosides A-C	Aqueous leaf and ethanolic extracts with curative anti-inflammatory activity attenuated paclitaxel toxicity in rat’s intestine	[54, 81, 82]
*Momordica* *foetida*	Shoot	Water	6.16 (NF54); 0.35 (FCR3)	Saponins, alkaloids, cardiac glycosides	No toxicity against human hepatocellular (HepG2) and urinary bladder carcinoma (ECV-304, derivative of T-24) cells	[83–85]
*Mondia* *whitei*	Whole root	MeOH	2% parasitemia suppression (ANKA)	Chlorogenic acid	Low toxicity on mice exposed to extract for 90 days	[86, 87]
*Moringa* *oleifera*	Flower, leaves	Water, MeOH, acetone, hexane	Parasitemia suppression upto 99.48%	Flavonoids	Not toxic	[88]
*Ocimum* *gratissimum*	Leaves/twigs	DCM	8.6 (W2)	Flavonoids	LD_50_ of the butanolic and ethyl acetate fraction of MeOH leaf extract were 2154.1 and 3807.9 mg/kg	[64, 89, 90]
*Ocimum* *kilimandscharicum*	Leaves, twigs	DCM	0.843 (D6); 1.547 (W2)	No reports	No reports	[89]
*Plectranthus* *barbatus*	Leaves	DCM	No activity	No toxicity recorded	No reports	[89, 91]
	Rootbark	Water (hot), chloroform/MeOH	100 mg/kg/day of extracts had 55.23% and 78.69% parasite chemosuppression	No report	No reports	
*Senna* *didymobotrya*	Leaves twigs	MeOH, DCM/MeOH (1:1)	> 100 (K39), 9.5 (D10)	Quinones	Root extracts were toxic (LD_50_ = 1927 mg/kg)	[55, 69, 92]
*Solanecio* *mannii*	Leaves	MeOH	21.6 (3D7), 26.2 (W2)	Phytosterols, n-alkanes and N-hexacosanol	No reports	[36, 93]
*Tithonia* *diversifolia*	Aerial parts, leaves	MeOH, ether	1.2 (3D7); 1.5 (W2), MeOH extract had 74% parasitemia suppression	Tagitinin C, sesquiterpene lactones	Aerial parts reportedly cytotoxic against cells from human foetal lung fibroblast cell line	[94–97]
*Vernonia* *amygdalina*	Leaves	MeOH/DCM, ethanol	2.7 (K1), 9.83. In vivo parasite suppression of between 57.2–72.7% in combination with chloroquine	Vernolepin, vernolin, vernolide, vernodalin and hydroxy vernodalin, steroid glucosides	Petroleum ether extract elicited strong cytotoxicity	[36, 54, 57, 85, 95, 98]
*Warburgia* *ugandensis*	Stem bark	MeOH, water, DCM	6.4 (D6); 6.9 (W2), 12.9 (D6); 15.6 (W2) 69% parasite suppression	Coloratane sesquiterpenes, e.g., muzigadiolide	Cytotoxic to the human glioblastoma cell line U87 CD4 CXCR4 (​​CC_50_ = 7.2 and 2.0 μg/ml for ethanol and DMSO extracts	[60, 61, 95, 99–101]

^1^Solvents used: DCM = dichloromethane, MeOH = methanol. ^2^Antiplasmodial activity are reported as IC_50_ (μg/mL). *Plasmodium* *falciparum* isolates: chloroquine sensitive strains are 3D7, MRC-2, D6, D10, FCR3, and NF54; chloroquine resistant strains include DD2, K1 and W2

## Conclusion

The local communities in Bwambara sub-county of Rukungiri District possess rich and diverse indigenous knowledge of plant-based medicine used to manage malaria. This could indicate high malaria cases, and/or accessibility, availability and potency of herbal remedies. More than 30% of the plants recorded in this study have been reported elsewhere for treatment of malaria and have experimentally recorded antimalarial and antiplasmodial activities. No study has reported any pharmacological properties, safety and efficacy of *Digitaria* *abyssinica* and *Berkheya* *barbata*. There is need to validate the toxicity and efficacy of other unstudied species to foster the discovery of efficacious, safe and effective standardized antimalarial drugs.

### Supplementary Information

**Additional file 1.** Details of medicinal plants used in the management of malaria in Bwambara Subcounty, Rukungiri District, Uganda.

## Data Availability

The raw data supporting the conclusions of this study are available within this article and its supplementary files.

## Abbreviations

DCM: Dichloromethane
ICF: Informant consensus factor
FC: Frequency of citation
ITN: Insecticide-treated mosquito nets
MeOH: Methanol
WHO: World Health Organization
