# Supplementary material for: Ethnomedicinal plants used for malaria treatment in Rukungiri District, Western Uganda
Source: Trop Med Health. 2023 Aug 30;51:49. doi: 10.1186/s41182-023-00541-9 (PMC10466780; doi:10.1186/s41182-023-00541-9)
Supplement: Supplementary file 1 — Additional file 1. Details of medicinal plants used in the management of malaria in Bwambara Subcounty, Rukungiri District, Uganda. [file 41182_2023_541_MOESM1_ESM.docx]

**Supplementary file: Additional File S1.** Details of medicinal plants used in the management of malaria in Bwambara Subcounty, Rukungiri District, Uganda

| **No.** | **Family**  *Botanical name* | **Voucher no.** | **Local name** | **Part used** | **Life form** | **Mode of Preparation Administration** | **FIC** | **UV** |
| --- | --- | --- | --- | --- | --- | --- | --- | --- |
|  | **Acanthaceae** |  |  |  |  |  |  |  |
| 1 | *Justicia anselliana* (Nees) T. Anderson | H22-013 | Kwiniini | L | Shrub | Decoction taken (3 spoonful twice daily for adults and half teaspoonful twice daily for children) | 1 | 0.008 |
|  | **Apocynaceae** |  |  |  |  |  |  |  |
| 2 | *Mondia whitei* (Hook.f.) Skeels | H22-030 | Omurondo | L | Vine | Cold infusion taken (1 glass) | 1 | 0.008 |
| 3 | *Cascabela thevetia* (L.) Lippod | H22-049 | Musenene | L | Tree | Decoction with fresh leaves of *S. elliptica* and *T. peruviana* taken (1 cup twice times daily) | 1 | 0.008 |
|  | **Asphodelaceae** |  |  |  |  |  |  |  |
| 4 | *Aloe vera* (L.) Burm.f. | H22-022 | Rukaka | L | Herb | Decoction taken (1 glass for adults and ¼ glass for children). Decoction with crushed stem bark of *C. papaya*, and stems of *P. purpureum* and *S. officinarum* drunk (1 glass). Cold infusion and taken (1 cup once a day for two days) | 35 | 0.28 |
|  | **Asteraceae** |  |  |  |  |  |  |  |
| 5 | *Baccharoides lasiopus* (O.Hoffm) H.Rob. | H22-008 | Omujuma | L, RB | Shrub | Decoction taken (1 cup). Cold infusion and taken (half a glass)/Decoction of roots with leaves taken (1 cup) | 6 | 0.048 |
| 6 | *Berkheya barbata* (L.f.) Hutch. | H22-040 | Orugyembagyembe | L | Herb | Decoction with fresh leaves of *C. anisata* and *V. amygdalina*, and drink a teaspoon 2 times daily for children and a spoonful 3 times daily for adults | 1 | 0.008 |
| 7 | *Bidens pilosa* L. | H22-035 | Enyabarashana | Fl | Herb | Decoction with root bark of *V. amygdalina*, flesh leaves of *D. abyssinica* and flowers of *B. pilosa* and drink as tea 1 cup daily for 2 days. Cold infusion and drink half a glass for adults and 2 spoonsful for children | 3 | 0.024 |
| 8 | *Bothriocline longipes* (Oliv. & Hiern) N.E.Br. | H22-046 | Ekyoganyanja | L | Herb | Decoction with fresh leaves *V. amygdalina*, *P. barbatus* and *Aloe vera* and drink 1 glass. Decoction with fresh leaves of *M. foetida* and drink 1 cup | 1 | 0.008 |
| 9 | *Crassocephalum vitellinum* (Benth.) S. Moore | H22-016 | Esunuunu | L | Herb | Decoction drunk; half a glass every morning. Decoction with fresh leaves of *V. amygdalina*, *C. limonum* and *I. arrecta*, and bathe | 1 | 0.008 |
| 10 | *Vernonia amygdalina* Delile | H22-002 | Omubirizi | L, RB | Shrub | Cold infusion taken (1 cup 3 times daily for 7 days). Decoction (1 glass daily for 5 days)/ with a mixture of fresh leaves and stem bark (1 glass twice daily for 2 days)/with rock salt and drink a third of a cup 3 times daily for adults and a sixth of a cup for children taken/with fresh leaves of *C. vitellinum*, *C. limonum* and *I. arrecta*, and used for bathing | 76 | 0.608 |
| 11 | *Hoffmannanthus abbotianus* (O.Hoffm.) H.Rob., S.C.Keeley & Skvarla | H22-019 | Omuhe | L | Herb | Cold infusion taken (1 glass) or with water that stayed overnight (*amaizi g’endengwa*), take a full cup and cover self | 4 | 0.032 |
| 12 | *Laggera alata* (D.Don) Sch.Bip. ex Oliv | H22-020 | Ekitabataabe | L | Herb | Cold infusion taken (1 glass) | 1 | 0.008 |
| 13 | *Solanecio mannii* (Hook.f.) C. Jeffrey | H22-005 | Omukoona/omugango | L | Shrub | Cold infusion taken (1 cup twice daily) | 2 | 0.016 |
| 14 | *Sonchus oleraceus* L. | H22-011 | Ekizimyamuriro | Sht | Herb | Decoction with other thornless herbs and steam bath | 5 | 0.04 |
| 15 | *Tithonia diversifolia* (Hemsl.) A.Gray | H22-050 | Ngaroitaano | L | Shrub | Decoction taken (1 glass) or boiled and used to bathe when cold | 5 | 0.04 |
|  | **Bignoniaceae** |  |  |  |  |  |  |  |
| 16 | *Kigelia africana* (Lam.) Benth. | H22-036 | Omwefuzo | L | Tree | Cold infusion with leaves of *V. brachycalyx* taken (half a glass) | 1 | 0.008 |
| 17 | *Markhamia lutea* (Benth.) K.Schum | H22-031 | Omusavu | RB | Tree | Decoction with fresh leaves of *I. cylindrica*, *D. abyssinica* and root bark of *M. lutea* taken half a cup thrice daily | 1 | 0.008 |
|  | **Canellaceae** |  |  |  |  |  |  |  |
| 18 | *Warburgia ugandensis* Sprague | H22-014 | Omwiha | SB | Tree | Decoction taken (2 teaspoonful thrice daily. Half teaspoonful of cold infusion taken | 3 | 0.024 |
|  | **Caricaceae** |  |  |  |  |  |  |  |
| 19 | *Carica papaya* L. | H22-047 | Ekipapari | SB, L, Fr | Tree | Decoction with leaves of *Aloe vera* and *V. amygdalina* taken (1 glass). Cold infusion and taken (1 cup) | 6 | 0.048 |
|  | **Cleomaceae** |  |  |  |  |  |  |  |
| 20 | *Cleome gynandra* L. | H22-001 | Eshogyi | L | Herb | Cold infusion taken (half a glass) | 1 | 0.008 |
|  | **Cucurbitaceae** |  |  |  |  |  |  |  |
| 21 | *Momordica foetida* Schumach. | H22-025 | Omwihura | L, SB | Vine | Decoction with fresh leaves of *B. longipes* taken (1 cup).  Cold infusion and drink 1 glass | 2 | 0.016 |
|  | **Euphorbiaceae** |  |  |  |  |  |  |  |
| 22 | *Manihot esculenta* Crantz. | H22-033 | Muhogo | L | Shrub | Half a cup of cold infusion taken | 1 | 0.008 |
| 23 | *Shirakiopsis elliptica* (Hochst.) Esser | H22-009 | Omushasha | L | Tree | Decoction with fresh leaves of *T. peruviana* taken (1 cup) | 1 | 0.008 |
|  | **Fabaceae** |  |  |  |  |  |  |  |
| 24 | *Albizia coriaria* Welw. ex Oliv. | H22-012 | Omusisa | SB | Tree | Decoction with the stem bark of *M. foetida* taken (2 teaspoonful thrice daily) | 2 | 0.016 |
| 25 | *Erythrina abyssinica* Lam. | H22-010 | Ekiko | L | Tree | Decoction with fresh leaves of *V. amygdalina* taken (1 cup) | 3 | 0.024 |
| 26 | *Indigofera arrecta* Hochst. ex A.Rich. | H22-006 | Omusoroza | L, RB | Shrub | Decoction of fresh roots and leaves taken (2 glasses). Decoction with fresh leaves of *V. amygdalina* and *C. vitellinum* and bathe | 1 | 0.008 |
| 27 | *Senna didymobotrya* (Fresen.) H.S. Irwin & Barneby | H22-037 | Omugabagaba | L | Shrub | Boil fresh leaves and bathe. Decoction taken (1 glass) | 2 | 0.016 |
| 28 | *Vachellia hockii* (De Wild.) Seigler & Ebinger | H22-041 | Akagando | SB | Tree | Decoction taken (1 glass thrice daily) | 1 | 0.008 |
|  | **Lamiaceae** |  |  |  |  |  |  |  |
| 29 | *Clerodendrum capitatum* (Wild.) Schumach. | H22-021 | Ekyishekashekye | L | Shrub | Decoction drunk (1 glass) or cold infusion taken (1 cup) | 5 | 0.032 |
| 30 | *Ocimum gratissimum* | H22-003 | Omujaaja | L | Shrub | Decoction with the fresh leaves of *V. amygdalina* taken (1 glass) | 1 | 0.008 |
| 31 | *Ocimum kilimandscharicum* Gürke | H22-018 | Obushonga | L | Shrub | Decoction taken (1 glass) and/or used for a bathe. Cold infusion with the fresh leaves of *V. amygdalina* and *E. grandis*, add water, sieve and drink 1 glass | 2 | 0.016 |
| 32 | *Plectranthus barbatus* Andrews | H22-007 | Ekicuncu | L | Shrub | Decoction with the fresh leaves of *V. amygdalina*, *B. longipes* and *Aloe vera* taken (half a cup). Cold infusion taken (half a cup) | 3 | 0.024 |
|  | **Malvaceae** |  |  |  |  |  |  |  |
| 33 | *Sida alba* L. | H22-044 | Omucundeezi | L | Shrub | Decoction with fresh leaves of *Aloe vera* taken/cold infusion taken (1 glass) | 3 | 0.024 |
|  | **Meliaceae** |  |  |  |  |  |  |  |
| 34 | *Azadirachta indica* A. Juss. | H22-048 | Niimu | L | Tree | Decoction with fresh leaves of *Aloe vera* taken (1 glass thrice daily). Dry in shade, always take in tea or any drink for prevention of malaria. Cold infusion prepared and taken (half a cup twice daily) | 15 | 0.12 |
|  | **Moringaceae** |  |  |  |  |  |  |  |
| 35 | *Moringa oleifera* Lam. | H22-027 | Moringa | Seed, L | Tree | Chewed or Cold infusion of seeds taken (1 glass twice daily). Decoction taken (1 glass thrice daily) | 4 | 0.032 |
|  | **Musaceae** |  |  |  |  |  |  |  |
| 36 | *Musa acuminata* Colla | H22-023 | Omutumba | L | Herb | Cold infusion of yellowed leaves taken (half a cup) | 1 | 0.008 |
|  | **Myrtaceae** |  |  |  |  |  |  |  |
| 37 | *Eucalyptus grandis* W. Hill ex Maiden | H22-039 | Karutusi | L | Tree | Decoction taken (half a cup). Cold infusion with leaves of *V. amygdalina* and *O. kilimandscharicum* taken (1 glass) | 2 | 0.016 |
|  | **Poaceae** |  |  |  |  |  |  |  |
| 38 | *Digitaria abyssinica* (Hochst. ex A.Rich.) Stapf | H22-026 | Orumbugu | L | Grass | Decoction taken (1 glass thrice daily for adults and 1 teaspoonful thrice daily for children) | 6 | 0.048 |
| 39 | *Imperata cylindrica* (L.) P.Beauv. | H22-042 | Omushojwa | L | Grass | Decoction with leaves of *D. abyssinica* and root bark of *M. lutea* taken (half a cup thrice daily) | 1 | 0.008 |
| 40 | *Pennisetum purpureum* Schumach. | H22-029 | Ekibingo | St | Grass | Decoction with crushed leaves of *Aloe vera*, stems of *C. papaya* and *S. officinarum* taken (1 glass) | 2 | 0.016 |
| 41 | *Saccharum officinarum* L. | H22-004 | Ekyikwijo | St | Grass | Decoction taken (1 glass) | 1 | 0.008 |
|  | **Rhamnaceae** |  |  |  |  |  |  |  |
| 42 | *Gouania longispicata* Engl. | H22-028 | Omufurura | L | Liane | Cold infusion taken (1 cup) | 1 | 0.008 |
|  | **Rutaceae** |  |  |  |  |  |  |  |
| 43 | *Citrus limon* (L.) Burm.fil. | H22-043 | Endimu | L, SB | Tree | Decoction taken (1 glass) or used for bathing | 2 | 0.016 |
| 44 | *Clausena anisata* (Willd.) Hook.f. | H22-032 | Omutanu | L | Shrub | Decoction taken (1 teaspoonful 2 times daily for children and 1 spoonful 3 times daily for adults) | 1 | 0.008 |
|  | **Solanaceae** |  |  |  |  |  |  |  |
| 45 | *Nicotiana tabacum* L. | H22-015 | Etaabe | L | Herb | Cold infusion with fresh leaves *C. capitatum* sieved and taken (1 glass) | 1 | 0.008 |
| 46 | *Physalis peruviana* L. | H22-034 | Ekituutu | L, RB | Herb | Cold infusion taken (1 glass) | 3 | 0.024 |
|  | **Verbenaceae** |  |  |  |  |  |  |  |
| 47 | *Lantana trifolia* L. | H22-024 | Omuhukye | L | Shrub | Cold infusion taken (1 glass) | 3 | 0.024 |
|  | **Vitaceae** |  |  |  |  |  |  |  |
| 48 | *Cyphostemma adenocaule* (Steud. ex A.Rich.) Desc. | H22-017 | Ekibombo | L | Liane | Decoction taken (1 cup) | 1 | 0.008 |
| 49 | Unidentified | NA | Rushariira | L | NA | Decoction taken (1 cup) | 6 | 0.048 |
| 50 | Unidentified | NA | Ekikoyooyo | L | NA | Decoction taken (1 cup) or used for bathing | 3 | 0.024 |
| 51 | Unidentified | NA | Ngaromwenda | L | NA | Cold infusion taken (1 cup) | 1 | 0.008 |
| 52 | Unidentified | NA | Kororokwini | L | NA | Decoction taken (1 cup) | 2 | 0.016 |
| 53 | Unidentified | NA | Dokita | L | NA | Decoction taken (half a cup twice daily) | 4 | 0.032 |
| 54 | Unidentified | NA | Ekikambakamba | L | NA | Decoction taken (1 cup) | 1 | 0.008 |
| 55 | Unidentified | NA | Ekitabaata | L | NA | Decoction with fresh leaves of *V. amygdalina* taken (1 cup) | 1 | 0.008 |

**Note**: NA = Not applicable, FC = frequency of citation, UV = Use Value, plant parts: Bk = Bark, Fl = Flowers, Fr = Fruits, L = Leaf, RB = Root bark, St = stem, SB = stem bark.
